# What are the risk factors for admission to the pediatric intensive unit among pediatric patients with COVID-19?

**DOI:** 10.1186/s13052-021-01057-w

**Published:** 2021-05-03

**Authors:** Susanna Esposito, Fabio Caramelli, Nicola Principi

**Affiliations:** 1 Pediatric Clinic, Department of Medicine and Surgery, University Hospital, University of Parma, Pietro Barilla Children’s Hospital, Via Gramsci 14, 43126 Parma, Italy; 2 Paediatric Intensive Care Unit, Scientific Institute for Research and Healthcare (IRCCS) Azienda Ospedaliero-Universitaria di Bologna, Bologna, Italy; 3 Università degli Studi di Milano, Milan, Italy

**Keywords:** Children, COVID-19, Pediatric intensive care unit, SARS-COV-2

## Abstract

**Background:**

Although with exceptions, evidence seems to indicate that children have lower susceptibility than adults to severe acute respiratory syndrome coronavirus 2 (SARS-CoV-2) infection. When infected, children generally remain asymptomatic or develop mild disease. A small number of pediatric cases required admission to the pediatric intensive care unit (PICU), respiratory support with a mechanical ventilation and additional life-saving interventions. Even if rarely, death can occur. Aim of this manuscript is to highlight the risk factors associated with severe outcome among pediatric patients with COVID-19.

**Main findings:**

Early identification of SARS-CoV-2-infected children at risk of developing severe COVID-19 is vital for service planning, as severely affected pediatric patients require high-quality care and should be followed only where an adequately structured PICU is available. However, early identification of children who must be carefully monitored for substantial risk of severe COVID-19 remains difficult. An underlying comorbidity and heart involvement are frequently observed in severe paediatric cases. Reduced left ventricular systolic function with an ejection fraction < 60%; diastolic dysfunction; and arrhythmias, including ST segment changes, QTc prolongation, and premature atrial or ventricular beat, are the earliest manifestations of heart involvement. Inclusion of heart enzyme serum levels and evaluation of ventricular function among predictive markers could lead to a more effective evaluation of children at risk with proper selection of those to admit to the PICU and with more adequate treatment in case of more severe clinical manifestations.

**Conclusions:**

To appropriately manage severe pediatric COVID-19 cases, greater attention should be paid to risk factors in children and adolescents, especially to cardiovascular alterations (e.g., heart enzyme serum levels and evaluation of ventricular function). Further studies are needed and the development of a validated score based on all the most common presumed markers of disease severity seems essential.

## Background

Although with exceptions, evidence seems to indicate that children have lower susceptibility than adults to severe acute respiratory syndrome coronavirus 2 (SARS-CoV-2) infection [1–3]. When infected, children generally remain asymptomatic or develop mild disease [4]. A joint report from the American Academy of Pediatrics and the Children’s Hospital Association indicated that on January 28, 2021, a total of 2,816,775 COVID-19 cases in children have been reported in the USA; a value that represents only 12.8% of cases in the USA and 1.2–2.9% of hospitalizations associated with the disease [5]. In the reporting states, child mortality accounted for 0.00–0.21% of COVID-19 deaths [5]. A small number of pediatric cases required admission to the pediatric intensive care unit (PICU), respiratory support with a mechanical ventilation and additional life-saving interventions. Even if rarely, death can occur. A multinational, multicenter cohort study carried out in Europe showed that among 582 children with laboratory-confirmed SARS-CoV-2 infection and a median age of 5.0 years, 62% were hospitalized, 8% required admission to the PICU, 5% had radiological findings suggestive of acute respiratory distress syndrome and 4% were intubated and mechanically ventilated [6]. Fatal outcomes occurred in 4 cases [6]. In a recent multicentre Italian study aimed at investigating epidemiological, clinical and therapeutic aspects of SARS-CoV-2 infection in infants, children and adolescents, 149/759 children (19.6% of the entire study population and 38.8%, 140/361, of those hospitalized for COVID-related causes) developed one or more complications [7]. Mean age was significantly higher among children with complications than in uncomplicated infections (6.9 vs. 5.5 years, respectively; *p* = 0.023), while gender was non-contributory to complications development. At multivariate analysis, fever or abdominal pain at onset and co-morbidities were risk factors for complications. Thirty children (4.0%) required intensive care support for COVID-19. Viral co-infections, underlying clinical conditions and age between 5 and 9 years were statistically related to PICU admission [7]. No information is available on chronic diseases most at risk of a serious course of the disease. No association was found with gender, ethnicity and socio-economic factors. Differences between the studies in hospitalization and PICU admission rates seem related mainly to protocols or clinical practice and not necessarily to disease severity. As regards hospitalized pediatric cases and those admitted to PICU, given the scarcity of data available in the literature, we recently highlighted that it would be advisable for pediatricians to identify a set of variables to be monitored over time using a shared methodology and definitions [8]. Aim of this manuscript is to highlight the risk factors associated with severe outcome among pediatric patients with COVID-19.

## Importance of early identification of pediatric patients with COVID-19 at risk of severe outcome

Early identification of SARS-CoV-2-infected children at risk of developing severe COVID-19 is vital for service planning, as severely affected pediatric patients require high-quality care and should be followed only where an adequately structured PICU is available. Hospitalization of children at risk in properly equipped units before the development of severe clinical manifestations seems essential to improve disease outcome, reducing the need for invasive treatment, duration of hospital stay, and risk of death [6].

## Risk factors associated with severe outcome among pediatric patients with COVID-19

In adults with COVID-19, it has been observed that independent risk factors associated with mortality included older age, male sex, high fraction of inspired oxygen, high positive end-expiratory pressure, and history of chronic obstructive pulmonary disease, hypercholesterolemia, and type 2 diabetes [9, 10]. Studies that have evaluated which factors could predict severe pediatric COVID-19 have shown that younger age, pre-existing underlying chronic severe comorbidities, male sex and lower respiratory tract infection signs and symptoms at presentation could be considered risk factors for PICU admission [11–13]. Among laboratory markers, increased leukocyte count, lymphopenia, and elevated inflammatory markers (C-reactive protein and procalcitonin) were those more frequently associated with PICU admission [11–13]. Only in some cases were lactate dehydrogenase, pro-B-type natriuretic peptide and troponin studied and found to be increased, suggesting a potential role of these variables as markers of risk.

Despite these findings, early identification of children who must be carefully monitored for substantial risk of severe COVID-19 remains difficult. Definitive conclusions about the real role of the suggested risk markers as predictors of severity cannot be drawn, as most of the studies have enrolled few patients [6–8]. Additionally, the criteria used for enrollment and evaluation differed significantly. The lack of a cutoff level for some parameters does not allow us to establish which patients are truly at risk. Regarding the role of age, there were large differences among studies. In some cases, it was reported that children ≤1 year old were at risk, whereas in others, only children ≤1 month old had a greater probability of developing severe disease [6–8]. Most cases of multisystem inflammatory syndrome in children (MIS-C), one of the most severe clinical manifestations of pediatric COVID-19, occur in school-age patients and have marginal epidemiological importance in younger children [14]. The presence of an underlying chronic disease in children admitted to the PICU has been documented in several studies and is generally considered the most reliable marker of severe COVID-19 in pediatrics [6]. However, the prevalence of these factors among PICU patients varies significantly. In some studies, the prevalence of comorbidities was documented in more than 70% of children with severe disease [15]. In a European study, only 52% of PICU cases had an underlying disease [6]. This conflicting evidence suggests that, if an underlying comorbidity may be a risk factor for severe COVID-19 in children, it is not the only factor capable of favoring the worsening of the clinical picture and the development of complications.

More effective identification of at-risk children could be obtained if several variables could be simultaneously considered, and a risk score could be developed. A similar solution was found to be effective in adults by Liang et al. [16] Starting with 72 variables characterizing COVID-19 adult patients, they identified 10 independent predictors of severe or critical disease. With a mathematical model, a well-defined value was attributed to each variable, and a score was developed [16]. Later, it was validated to show high efficacy in predicting a patient’s risk of developing critical illness. However, to prepare a pediatric score, criteria for admission to the PICU must be clarified, including the degree of respiratory impairment and cutoff levels of laboratory tests. The inclusion of potential markers of severity to date have been poorly considered, must be evaluated. Among these is heart involvement [16]. Several findings seem to indicate that, in the first phases of severe COVID-19, signs and symptoms of heart lesions can occur and be useful to select children at risk for complications [13, 17, 18]. A great number of previously healthy children with MIS-C present with cardiovascular alterations [19–21]. Some of them already have signs and symptoms of heart dysfunction in the first phases of disease, and it has been reported that they increase the need for PICU admission, development of potential long-term problems, and risk of death [13, 17, 18]. Reduced left ventricular systolic function with an ejection fraction < 60%; diastolic dysfunction; and arrhythmias, including ST segment changes, QTc prolongation, and premature atrial or ventricular beat, were the earliest manifestations. Moreover, increased troponin levels have been repeatedly reported in children with COVID-19 admitted to the PICU [13, 18, 19]. Inclusion of heart enzyme serum levels and evaluation of ventricular function among predictive markers could lead to a more effective evaluation of children at risk with proper selection of those to admit to the PICU and with more adequate treatment in case of more severe clinical manifestations.

## Conclusions

The criteria for admission to a PICU for the SARS-CoV-2-positive patient are not different from the general admission criteria for non-positive pediatric patients and are represented by the direct consequences of the viral infection (generally severe respiratory failure), by the complications of the infection (e.g., MIS-C) and by the onset, concomitantly with SARS-CoV-2 infection, of other conditions for which hospitalization is indicated [8, 20]. In addition, it will also be necessary to ensure a specific intensive track that ensures proper isolation, as well as the adoption of the appropriate safety devices by patients and their caregivers, in order to avoid dangerous cross-infections between patients and patients, and between patients and health care workers, according to international standards redefined on the basis of local policies [21]. Table 1 summarizes the recommendations that we developed for considering admission to the PICU in the Emilia-Romagna Region, Italy. To appropriately manage severe pediatric cases, greater attention should be paid to risk factors in children and adolescents, especially to cardiovascular alterations (e.g., heart enzyme serum levels and evaluation of ventricular function). Further studies are needed and the development of a validated score based on all the most common presumed markers of disease severity seems essential.

**Table 1:** Recommendations for considering admission to the pediatric intensive care unit (PICU) in the Emilia-Romagna Region, Italy

**When is it recommended to admit a pediatric patient to the PICU?**	
In case of the need for CPAP in the COVID-positive patient in acute respiratory failure, hospitalization in a referral center equipped with a PICU is recommended.	
Hospitalization in the PICU is recommended in case of the following:	
•Tachypnea associated with at least one of the following signs or symptoms: dyspnea, elevated work of breathing, cyanosis, hypoxia and/or desaturation, inability to eat and drink, lethargy, unconsciousness or convulsions;	
•Acute respiratory distress syndrome (PaO2/FiO2 ≤ 300 in bilevel or CPAP ≥5 cm/H_2_O);	
•Need for inotropic support and/or hemodynamic monitoring;	
•Instability, cardiocirculatory insufficiency or any condition of shock;	
•Multiorgan failure;	
•Severe sepsis;	
•Coma.	
**What are the risk factors for admission to the PICU?**	
•Presence of new onset left ventricular dysfunction, coronary abnormalities or pericardial effusion on echocardiography;	
•Deterioration of laboratory tests (C reactive protein, procalcitonin, interleukin-6, ferritin, D-dimer, troponin, liver indices, and lactate dehydrogenase);	
•Progressive respiratory failure: failure to reduce heart rate, respiratory rate and work of breathing and failure to improve O_2_ saturation, 30 min and 2 h from the start of high flow therapy are highly predictive of noninvasive ventilation failure, as well as elevated PRISM values basal, CO_2_ and FiO_2_.	

*CPAP* Continuous Positive Airway Pressure, *PRISM* Pediatric Risk of Mortality Score

## Data Availability

All included.

## Abbreviations

COVID-19: New coronavirus disease 2019
CPAP: Continuous Positive Airway Pressure
MIS-C: Multisystem inflammatory syndrome in children
PICU: Pediatric Intensive Care Unit
PRISM: Pediatric Risk of Mortality Score
SARS-CoV-2: Severe acute respiratory syndrome coronavirus
